# A pilot study exploring the relationship between lifelong learning and factors associated with evidence-based medicine

**DOI:** 10.5116/ijme.576f.a2ca

**Published:** 2016-07-03

**Authors:** Misa Mi, Alexandra Halalau

**Affiliations:** 1 Department of Biomedical Sciences, Oakland University William Beaumont School of Medicine, Rochester, Michigan, USA; 2 Department of Internal Medicine, Oakland University William Beaumont School of Medicine, Royal Oak, Michigan, USA

**Keywords:** Evidence-based medicine, information management skill, learning environment, lifelong learning, medical residents, self-efficacy

## Abstract

**Objectives:**

To explore possible relationships between residents’ lifelong learning orientation, skills in practicing evidence-based medicine (EBM), and perceptions of the environment for learning and practicing EBM.

**Methods:**

This was a pilot study with a cross-sectional survey design. Out of 60 residents in a medical residency program, 29 participated in the study. Data were collected using a survey that comprised three sections: the JeffSPLL Scale, EBM Environment Scale, and an EBM skill questionnaire. Data were analyzed using SPSS and were reported with descriptive and inferential statistics (mean, standard deviation, Pearson’s correlation, and a two-sample t-test).

**Results:**

Mean scores on the JeffSPLL Scale were significantly correlated with perceptions of the EBM Scale and use of EBM resources to keep up to date or solve a specific patient care problem. There was a significant correlation between mean scores on the EBM Scale and hours per week spent in reading medical literature to solve a patient care problem. Two-sample t-tests show that residents with previous training in research methods had significantly higher scores on the JeffSPLL Scale (p=0.04), EBM Scale (p=0.006), and self-efficacy scale (p =0.024).

**Conclusions:**

Given the fact that physicians are expected to be lifelong learners over the course of their professional career, developing residents’ EBM skills and creating interventions to improve specific areas in the EBM environment would likely foster residents’ lifelong learning orientation.

## Introduction

Lifelong learning has been regarded as a vital skill for any physician committed to providing current, safe and high-quality medical care to individual patients.^1^ Lifelong learning is defined as “an attribute involving a set of self-initiated activities and information-seeking skills with sustained motivation to learn and the ability to recognize one’s own learning needs”.^2^^,^^3^ For healthcare providers, they are expected to engage in lifelong learning due to the nature of their work--dealing with human life, meeting patients’ healthcare needs--in an environment where knowledge, technology, and social requirements are rapidly and continuously changing.^4^

In graduate (or postgraduate) medical education, it is required that training programs be developed based on ACGME competencies (Accreditation Council for Graduate Medical Education). One particular ACGME competency, practice-based learning and improvement, underscores the importance of lifelong learning, continuing professional development, and evidence-based practice. To achieve the competency, residents need to develop a new set of skills in self-reflecting and evaluating their care of patients, identifying patient care needs, translating the needs to clear and focused clinical questions, locating and appraising clinical research evidence, and integrating the evidence into patient care efforts.

There has been an increasing emphasis on quality improvement in health care, patient satisfaction, and integration of evidence-based approaches in patient care to ensure high-quality patient outcomes. With evidence-based practice gaining ground since its introduction in the early 90’s,^5^ EBM has been advocated as an approach to improving patient care outcomes.^6^ EBM is viewed as “a process of life-long, self-directed learning in which caring for our own patients creates the need for clinically important information about diagnosis, prognosis, therapy, and other clinical and health care issues”.^7^ It should be pointed out that new scientific developments in medicine and the exponential growth in biomedical literature have posed challenges for physicians to locate clinically relevant information and stay updated with the current literature as lifelong learners. Clearly, it is necessary to identify ways to enhance physicians’ lifelong learning so that they can constantly update their approaches to patient care and become evidence-based practitioners.

Over the past decade, EBM has been widely integrated into residency training programs. Nevertheless, it remains unknown as to how residents’ lifelong learning was related to factors associated with EBM such as information management skills, EBM activities, research training and experience, and the EBM learning environment. The purpose of the study was to investigate how residents’ orientation toward lifelong learning was associated with these factors and the EBM Learning Environment. It was anticipated that insights gleaned from the study would help program directors and clinical educators to identify ways to improve residents’ competency in practice-based learning and improvement and to make informed decisions on types of interventions to promote residents’ lifelong learning.

## Methods

### Study design

This was a pilot study utilizing a cross-sectional survey design.

### Participants

The study used a convenience sample of residents from the Internal Medicine Residency Program at the Beaumont Hospital on Royal Oak campus, Michigan. Out of 60 residents, 29 participated in the study by responding to a survey (a response rate of 48%). All participants were between the ages of 25-34, and 59% were male. The majority was PGY-1 (48%), and about half (45%) was from a medical school in the US.

### Data collection 

The survey that was administered to residents included three sections: the Revised Jefferson Scale of Physician Lifelong Learning (the JeffSPLL Scale, Medical Student Version),^8^ the EBM Environment Scale (EBM Scale),^9^ and an EBM skill self-assessment questionnaire.

The JeffSPLL Scale is a 14-item instrument to measure students’ lifelong learning orientation in medical education. Participants indicate their agreement or disagreement with each statement on a 4-point Likert scale from 1 (strongly disagree) to 4 (strongly disagree). Higher scores indicate a stronger orientation toward lifelong learning.  The EBM Scale consists of 36 items and 7 subscales representing different dimensions or aspects of the EBM learning environment. Participants indicate how much they agree or disagree with each statement on a 5-point Likert scale ranging from 1 (strongly disagree) to 5 (strongly disagree). Lower scores on the scale signify less favorable perceptions and higher scores more favorable perceptions of the EBM environment. Psychometric analyses conducted in previous studies provided strong evidence of the internal structure, reliability, and validity for both instruments.^8^^,^^9^ The last section of the survey, the EBM skill self-assessment questionnaire, consisted of items identified from a review of the literature and adapted from previous research.^10^ It comprised the following components that assessed information management and other EBM skills:

   •   Questions 1-3 assessed residents’ habit of reading medical literature;

   •   Question 4 (InfoUse1), a 9-item scale, determined the frequency of residents’ use of various EBM resources for keeping up to date;

   •   Question 5 (InfoUse2), a 9-item scale, measured the frequency of residents’ use of various EBM resources for solving a specific patient care problem;

   •   Question 6, a 7-item self-efficacy scale, gauged residents’ efficacy belief in their skills in practicing EBM.

   •   Questions 7 asked about residents’ previous involvement/experience in research; questions 8-9 on previous training in critical appraisal, research methods, epidemiology, and statistics, all of which were considered relevant in learning and practicing EBM. The questionnaire also included four items on demographic information (age, gender, level of training in residency, and level of training in EBM).

### Procedure

A packet with the survey and informed consent was distributed to residents at the beginning of one of their daily lectures during their outpatient clinic rotation. The informed content explained the purpose of the study and the nature of the study with voluntary participation and anonymous data collection. Study participants had 30 minutes to complete the survey. Data were collected from September 2015 to December 2015. The study was conducted with the approval of the Human Investigation Committee of the Beaumont Hospital Research Institute.

### Data analysis

Data collected were checked for completeness and accuracy. Using SPSS (version 17), the obtained data were analyzed by

 using means, standard deviation, Pearson’s correlation coefficient and a two-sample t-test at the significant level of α = 0.05.

## Results

The average mean (±standard deviation) of the JeffSPLL Scale was 3.32±0.38. The average mean of the EBM Scale was 4.04±0.49. The three components of the EBM skill questionnaire (InfoUse1, InfoUse2 and self-efficacy) had average mean of 2.96±0.50, 2.95±0.57, and 3.44±0.63, respectively (Table 1).

**Table 1:** Mean scores and standard deviation for all scales, n=29

Variable	Mean (SD)
JeffSPLL Scale	3.32 (0.38)
EBM Learning Environment	4.04 (0.49)
InfoUse1	2.96 (0.50)
InfoUse2	2.95 (0.57)
Self-Efficacy	3.44 (0.63)

More than ½ (62%) respondents reported previous experience in conducting research. Their previous training in different topics varied: 55% reported having previous training in critical appraisal; 28% in research methods; 31% in epidemiology; and 45% in statistics. The internal consistencies (Cronbach alpha) of different scales ranged from 0.52 to 0.96.

### Relationships of the JeffSPLL Scale with other scales

The Pearson’s correlations were calculated to identify any relationships between the JeffSPLL Scale, EBM Scale, InfoUse1, InfoUse2, and self-efficacy scale (Table 2). The correlational analysis shows that the JeffSPLL Scale was significantly correlated with the EBM Scale (r=0.4, p =0.035), InfoUse1 (r=0.41, p =0.032), and InfoUse2 (r=0.49, p =0.009). However, no significant correlation was found between the JeffSPLL Scale and self-efficacy. As to the EBM Scale, it was not significantly correlated with InfoUse1, InfoUse2, and self-efficacy. A strong association was observed between InfoUse1 and InfoUse2 (r=0.71, p <0.001); while self-efficacy was moderately correlated with InfoUse1 and InfoUse2.

**Table 2:** Correlations among different scales

Variable	JeffSPLL Scale	EBM Scale	InfoUse1	InfoUse2
JeffSPLL Scale				
EBM Scale	0.40*			
InfoUse1	0.41*	0.03		
InfoUse2	0.49*	0.14	0.71*	
Self-Efficacy	0.19	0.03	0.28	0.33

*p<005

### Relationships of previous research experience or training with different scales

Appendix A displays the relationships between the questions 7, 8, 9a, 9b and 9c with the JeffSPLL Scale, EBM Scale, InfoUse1, InfoUse2, and self-efficacy scale. Two-sample t-tests were computed to compare differences between residents with previous research or training experience and those without such experience queried in questions 7-9c on the scales. As Appendix A illustrates, there were no significant differences in mean scores on the JeffSPLL Scale, EBM Scale, InfoUse1, InfoUse2, and self-efficacy regardless of previous research experience or training in critical appraisal. However, regarding previous training in research methods, participants with such training scored significantly higher on the JeffSPLL Scale (p=0.04), EBM scale (p=0.006), and self-efficacy scale (p =0.024). A significant difference was also observed in mean scores on the self-efficacy when comparing residents with previous training in epidemiology with those without such training. But no significant differences were found in scores on different scales with regard to previous training in statistics.

### Relationships of habits of reading medical literature with different scales

Correlation coefficients were calculated to identify any relationships of questions 1-3 (literature reading habits) with different scales. There was only one significant correlation between hours per week spent in reading medical literature to solve a patient care problem (Question 2b) and mean scores on the EBM Scale (r=0.44, p=0.018). Two-sample t-tests were calculated to compare differences between residents with previous research or training experience and those without such experience assessed in questions 7-9c on questions 1-3. No significant differences were observed in habits of reading medical literature on previous research experience, previous training in critical appraisal, research methods, and epidemiology. However, one significant difference was found in the average number of hours per week spent reading medical literature for browsing/keeping up to date (Question 2b) with respect to previous training in statistics. Those with training spent on average 2.1±1.4 hours while those without training spent 4.3±3.5 hours (p =0.041).

## Discussion

The current study was the first one that established the link between residents’ lifelong learning orientation and their perceptions of the EBM learning environment and information management skills. As the results of the study indicate, residents who had a strong lifelong learning orientation tended to perceive the EBM learning environment favorably, suggesting that an environment conducive to residents’ learning and practice of EBM would likely contribute to residents’ strong lifelong learning orientation.

For residents who had a strong lifelong learning orientation, they tended to use EBM resources more frequently to stay updated or solve a specific patient care problem. The finding suggests that residents’ use of EBM resources for the purpose of keeping up with the literature or solving patient problems could contribute to their lifelong learning  orientation. Thus, it can be hypothesized that equipping residents with skills in accessing and using EBM resources could enhance their EBM skills that are associated with their strong life-long learning orientation.

However, residents’ self-efficacy belief in EBM skills was not significantly correlated with their lifelong learning orientation and perceptions of the EBM environment. The finding may indicate that residents’ self-efficacy was independent of the influence of their lifelong learning orientation and the EBM environmental factors.

The results of the study show no significant association between residents’ information use and their previous training experience in research methods. However, residents with previous training in research methods tended to have a strong lifelong learning orientation, favorable perceptions of the EBM environment, and a strong sense of self-efficacy. There are several possible explanations for the strong association between these variables. First of all, training in research methods provides residents with knowledge of study designs and develops their ability to critique a research study or conduct a research project. As a result, such training experience could expand residents’ capability to pursue their individual learning goals and thus, improve their perceptions of the EBM environment. Second, the understanding of research methods may enable residents to scrutinize clinical research studies critically and to identify the best evidence for patient care. Furthermore, the understanding of research methods may boost residents’ efficacy beliefs in EBM skills that are important for lifelong learning. As their confidence in practicing EBM grows, their lifelong learning orientation and perceptions of the EBM environment can be affected in a positive way.

The study reveals that previous training in critical appraisal, epidemiology or statistics was not significantly correlated with residents’ lifelong learning orientation, perceptions of the EBM environment, or use of EBM resources. While residents with training experience in epidemiology tended to have a strong sense of self-efficacy in EBM skills, training in critical appraisal or statistics was not associated with their information efficacy beliefs. A critical review of graduate medical education curricula in EBM reveals an emphasis on critical appraisal skills,^11^ but a research synthesis on the effectiveness of instruction in critical appraisal confirms that there is no evidence of increased use of the literature by simply learning critical appraisal skills.^12^ Another interesting finding of the study was that previous training in statistics was associated with a lower number of hours per week spent reading medical literature for browsing/keeping up to date. The finding may be interpreted such that understanding or knowledge of statistics could potentially help residents achieve fluency or efficiency in reading clinical research studies or read more studies with fewer hours.

Given the strong relationship of previous training in research methods with residents’ lifelong learning orientation, perceptions of the EBM learning environment, and self-efficacy, basic research methods should be included as an integral part of EBM training programs so that residents understand the types of study design best to answer a specific clinical question. In addition, the understanding of basic statistical concepts is also necessary to build the fluency or develop the efficiency in reading medical literature. The inclusion of basic research methods and statistical concepts as learning objectives in EBM training may potentially foster residents’ lifelong learning orientation, shape their attitude toward learning and practicing EBM, affect their reading of medical literature, and develop a strong sense of efficacy beliefs in EBM skills.

The results of the study also demonstrate the strong association of residents’ weekly hours spent in reading medical literature for a patient care problem with their perceptions of the EBM environment. The finding indicates that the EBM learning environment may be an important determinant of residents’ reading habit within the context of solving an actual patient problem. Various conditions that constitute the EBM learning environment may include expectations for residents as learners, protected time allowed for learning and practicing EBM, mentoring and role-modeling, feedback provided, and the EBM practice culture.^9^ Reading medical literature for solving patient problems is more likely to create a sense of purpose and to motivate residents to engage in problem-based or inquiry-based learning within the context of patient care. Obviously, it is important for residency program directors and clinical educators to take into consideration the learning conditions and develop and implement educational strategies that begin with residents’ real-time clinical questions^11^ and integrate EBM into the clinical teaching of trainees and the flow of patient care.^13^

### Limitations

There are several limitations inherent in this study. First, the survey was administered to a convenience sample of medical residents at one residency training program. The sample may not represent the population for which the scale was intended. The results may not be generalizable to residents at other training sites or in other specialties. Therefore, further research is needed to investigate the relationship between all variables to increase the generalizability of findings to a larger population. Secondly, with a small sample size in the study, the correlations among the instruments are potentially subject to the influence of chance factors. It warrants further research with a larger sample of residents to examine how the degree of the influence of chance factors may change and affect the correlations among the instruments. Third, the survey relied on respondents’ self-report which was subject to personal or recall bias. Their impression and memory may not accurately reflect their EBM learning and practice environment, their attitude towards lifelong learning, or their actual information skills. Fourth, study participation was voluntary and participants were all self-selected, which may lead to biased responses. Therefore, data collected may not adequately represent those who chose not to participate in the study.

### Implications for EBM teaching and research

Previous research establishes the link between past academic performance and a lifelong learning orientation^14^ and between the orientation and future academic achievement.^1^ This study may serve as a starting point for further research on the impact of multifaceted factors or variables that are likely to facilitate the development of a strong lifelong learning orientation of physicians in training. These factors are about the EBM learning environment, reading habit, information management skills, and self-efficacy in EBM skills. The findings of the study have offered an additional perspective to the literature on graduate medical education on residents’ lifelong learning orientation associated with these variables. To the authors’ knowledge, this was the first empirical study that investigated the relationship between these variables that interact to affect residents’ lifelong learning orientation.

The findings suggest implications for EBM teaching and learning within the clinical context. Slawson and his colleagues emphasize the value and importance of information management skills for all residents and practicing physicians in learning and practicing evidence-based medicine. In their point of view, teaching information management skills will prepare residents and practicing physicians for a practice of medicine that requires lifelong learning.^15^ An information mastery approach to teaching EBM has proved to increase residents’ confidence in evaluating and using the evidence.^16^ The study yielded evidence demonstrating a strong lifelong learning orientation associated with the use of EBM resources and favorable perceptions of the environment for learning and practicing EBM. Therefore, building information management skills and assessing the EBM learning environment to identify specific areas for improvement should be considered viable approaches to promoting residents’ lifelong learning orientation.

In developing and implementing an EBM program for residents, program developers or clinical educators may need to consider literacy in research designs, basic concepts of epidemiology and statistics as part of instructional content to equip residents with the fundamentals of learning and practicing EBM. As a strong lifelong learning orientation may affect residents’ motivation to engage in EBM activities (e.g., reading the medical literature) for EBM practice, it is useful to integrate EBM learning into clinical practice by tying residents’ reading of medical literature with patient care problems.

## Conclusions

The findings of the study establish the strong links between residents’ lifelong learning orientation, their perceptions of the EBM environment, and information management skills. Given the fact that physicians are expected to be lifelong learners over the course of their professional career, EBM training should incorporate information management skills and literacy in research methods and also attend to the affective dimension of EBM skills (self-efficacy). Meanwhile, it is essential to assess the EBM learning environment to identify specific areas (contextual factors) for improvement, for the EBM environment serves to mediate the interactions of different factors that attribute to lifelong learning.

### Acknowledgements

The authors would like to thank Michelle Jankowski for her assistance with data analysis.

### Conflict of Interest

The authors declare that they have no conflict of interest.

## Supplementary material

Supplementary file 1 Appendix A. Survey instrument: attributes of health educators
