# Supplementary material for: A pilot study exploring the relationship between lifelong learning and factors associated with evidence-based medicine
Source: Int J Med Educ. 2016 Jul 3;7:214–9. doi: 10.5116/ijme.576f.a2ca (PMC4939218; doi:10.5116/ijme.576f.a2ca)
Supplement: Supplementary file 1 — Appendix A. Survey instrument: attributes of health educators [file ijme-7-214-S1.pdf]

Appendix A

Correlations of different scales with previous research experience and training

Scale Mean	Involvement in Research			Critical Appraisal			Research Methods			Epidemiology			Statistics		
	Yes (N= 18)	No (N= 11)	p	Yes (N= 16)	No (N= 13)	p	Yes (N= 8)	No (N= 21)	p	Yes (N= 9)	No (N= 20)	p	Yes (N= 13)	No (N= 16)	p
Lifelong Learning	3.3 ± 0.4	3.3 ± 0.3	0.582	3.3 ± 0.3	3.4 ± 0.4	0.668	3.1 ± 0.4	3.4 ± 0.3	0.04	3.1 ± 0.4	3.4 ± 0.3	0.067	3.2 ± 0.4	3.4 ± 0.3	0.327
EBM Scale	(4.0 ± 0.6	4.2 ± 0.3	0.265	4.1 ± 0.5	4.0 ± 0.5	0.704	3.6 ± 0.5	4.2 ± 0.4	0.006	3.8 ± 0.6	4.1 ± 0.4	0.143	3.9 ± 0.5	4.1 ± 0.4	0.225
InfoUse1	3.0 ± 0.3	2.9 ± 0.7	0.736	3.0 ± 0.4	2.9 ± 0.6	0.834	2.9 ± 0.3	3.0 ± 0.6	0.786	2.9 ± 0.3	3.0 ± 0.6	0.869	3.1 ± 0.4	2.8 ± 0.5	0.178
InfoUse2	3.0 ± 0.6	2.9 ± 0.6	0.959	3.0 ± 0.6	2.9 ± 0.5	0.741	2.8 ± 0.7	3.0 ± 0.5	0.456	2.8 ± 0.3	3.0 ± 0.6	0.396	3.0 ± 0.6	2.9 ± 0.6	0.916
Self-Efficacy	3.5 ± 0.7	3.3 ± 0.6	0.564	3.5 ± 0.7	3.4 ± 0.6	0.625	3.9 ± 0.5	3.3 ± 0.6	0.024	3.8 ± 0.4	3.3 ± 0.7	0.036	3.6 ± 0.5	3.3 ± 0.7	0.245
